# Baleen hormones: a novel tool for retrospective assessment of stress and reproduction in bowhead whales (*Balaena mysticetus*)

**DOI:** 10.1093/conphys/cou030

**Published:** 2014-08-12

**Authors:** Kathleen E. Hunt, Raphaela Stimmelmayr, Craig George, Cyd Hanns, Robert Suydam, Harry Brower, Rosalind M. Rolland

**Affiliations:** 1 John H. Prescott Marine Laboratory, Research Department, New England Aquarium, Boston, MA 02110, USA; 2 Department of Wildlife Management, North Slope Borough, PO Box 69, Barrow, AK 99723, USA; 3 Institute of Arctic Biology, University of Alaska Fairbanks, 902 N. Koyukuk Drive, PO Box 757000, Fairbanks, AK 99775-7000, USA

**Keywords:** Baleen, cortisol, progesterone, reproduction, stress, whales

## Abstract

Arctic marine mammals are facing increasing levels of many anthropogenic stressors. Novel tools are needed for assessment of stress physiology and potential impacts of these stressors on health, reproduction and survival. We have investigated baleen as a possible novel tissue type for retrospective assessment of stress and reproductive hormones. We found that pulverized baleen powder from bowhead whales (*Balaena mysticetus*) contained immunoreactive cortisol and progesterone that were detectable with commercially available enzyme immunoassay kits. Both assays passed parallelism and accuracy validations using baleen extracts. We analysed cortisol and progesterone at the base of the baleen plate (most recently grown baleen) from 16 bowhead whales of both sexes. For a subset of 11 whales, we also analysed older baleen from 10, 20 and 30 cm distal to the base of the baleen plate. Immunoreactive cortisol and progesterone were detectable in all baleen samples tested. In base samples, females had significantly higher concentrations of cortisol and progesterone compared with males. Cortisol concentrations in older baleen (10, 20 and 30 cm locations) were significantly lower than at the base and did not exhibit correlations with age-class or sex. Progesterone concentrations were significantly higher in females than in males at all baleen locations tested and were significantly higher in pregnant females than in non-pregnant females. Four of five mature females showed dramatic variation in progesterone concentrations at different locations along the baleen plate that may be indicative of previous pregnancies or luteal phases. In contrast, all males and all immature females had uniformly low progesterone. Baleen hormone analysis is a novel approach that, with further methodological development, may be useful for determining individual longitudinal profiles of reproductive cycles and stress responses.

## Abstract (precis)

Baleen samples from sixteen bowhead whales contained measurable cortisol and progesterone, and both hormones demonstrated significant correlations with sex, age class and reproductive state. Analysis of hormones in baleen shows promise as a novel tool for retrospective analysis of stress and reproductive physiology of large mysticete whales.

## Introduction

Arctic marine mammals are faced with increasing exposure to a variety of ecological and anthropogenic stressors, particularly climate change and associated increases in seismic exploration, oil and gas development, ship traffic and fishing activity (Doney *et al.*, 2011; Pompa *et al.*, 2011; Reeves *et al.*, 2011; Moore *et al.*, 2012). The potential impacts of these rapid environmental changes on stress physiology, reproductive physiology and related population-level effects are largely unknown. Moreover, such physiological responses are particularly challenging to assess in the case of mysticete whales (baleen whales), which are difficult to study with standard physiological techniques (Hunt *et al.*, 2013).

Assessment of steroid hormones in novel tissue types offers a potential solution. Conservation physiologists are increasingly exploring different tissue types, as diverse as urine, faeces, hair, blubber, respiratory vapour and earwax, to measure steroid hormones relevant for addressing questions of stress physiology (e.g. adrenal steroids) and reproductive physiology (e.g. oestrogens, progestins, androgens; Amaral, 2010; Hunt *et al.*, 2013; Kellar *et al.*, 2013; Trumble *et al.*, 2013). In whales, the progestin content of faeces and blubber has been used to diagnose pregnancy (Rolland *et al.*, 2005; Kellar *et al.*, 2006, 2013), and elevated faecal glucocorticoids have been shown to reflect exposure to various acute and chronic stressors (Rolland *et al.*, 2012; Hunt *et al.*, 2013).

Mysticete whales have a unique tissue type that has not previously been explored for steroid hormone analysis, i.e. baleen. Baleen is a stratified, cornified epithelial tissue consisting of long, overlapping, fringed plates that grow downward from the upper jaw (Haldiman and Tarpley, 1993; Bragulla and Homerger, 2009; Young, 2012), which collectively form the whale's filter-feeding apparatus (Haldiman and Tarpley, 1993; Werth, 2013). It has recently become clear that most cornified epidermal tissues in vertebrates contain detectable steroid hormones; this has been well demonstrated in human hair and fingernails, mammalian fur, bird feathers and shed snakeskin (Bortolotti *et al.*, 2008; Warnock *et al.*, 2010; Ashley *et al.*, 2011; D'Anna-Hernandez *et al.*, 2011; Lattin *et al.*, 2011; Bechshøft *et al.*, 2012; Berkvens, 2013). The steroid content of these cornified tissues appears to reflect circulating hormone levels accumulated during tissue growth, e.g. human hair cortisol content reflects adrenal activity of the previous few months, whereas feather corticosterone content reflects adrenal activity that occurred during feather molt (Warnock *et al.*, 2010; Lattin *et al.*, 2011; Sharpley *et al.*, 2011). The glucocorticoid content of these tissues correlates significantly with exposure to known stressors (Yamada *et al.*, 2007; Van Uum *et al.*, 2008; Fairbanks *et al.*, 2011; Skoluda *et al.*, 2012) and also with consequent physiological impacts, such as reduced growth and body condition (Lattin *et al.*, 2011; Macbeth *et al.*, 2012; Kennedy *et al.*, 2013), reduced investment in offspring (Fairhurst *et al.*, 2012), increased disease susceptibility (Pereg *et al.*, 2011) and mortality risk (Koren *et al.*, 2012). Most research to date has focused on the adrenal glucocorticoids, but preliminary results indicate that reproductive steroids are also measurable in cornified tissues (Koren *et al.*, 2012; Snoj *et al.*, 2012; Caslini, 2013).

Baleen steroid analysis may offer two unusual benefits as a conservation physiology tool. First, baleen grows continuously from the whale's upper palate; newer baleen is added gradually at the base, and the oldest baleen is steadily worn off at the distal end (Best and Schell, 1996; Lubetkin *et al.*, 2008; Young, 2012). In mature bowhead whales (*Balaena mysticetus*), for example, ∼15–20 cm of baleen is added per year, and a single plate may be 4 m long; thus, one baleen plate from a mature bowhead may represent a continuous physiological record of approximately the past 20–25 years (Lubetkin *et al.*, 2012; N. Lysiak, personal communication). (Immature animals have shorter baleen and faster baleen growth rate, and would be expected to have maximal baleen growth records of ∼10–15 years depending on age; Lubetkin *et al.*, 2012; N. Lysiak, personal communication). Baleen might therefore contain information on longitudinal profiles of past reproductive cycles and physiological events that occurred during the last decade or two of the whale's life. This is of particular interest for bowhead whales, in which reproductive cycles are still poorly understood; for example, current estimates of calving intervals are based on only three observed cases (Rugh *et al.*, 1992). A second potential advantage of baleen is that there exist historical archives of baleen plates that date back to the era of commercial whaling (Bockstoce, 1986), as well as modern archives of baleen collected from whales that were stranded or taken in aboriginal subsistence whaling. Steroids in cornified tissues often remain detectable in specimens stored long term; museum specimens of hair and feathers that are decades to centuries old have been found to have detectable steroid hormones that demonstrate physiologically relevant patterns (Webb *et al.*, 2010; Bechshøft *et al.*, 2012). Therefore, the possibility exists that baleen hormones could be used as a valuable tool for retrospective comparisons of steroid profiles in past vs. present-day populations, for example, comparison of inter-calving intervals (Webb *et al.*, 2010; Bechshøft *et al.*, 2012; Kennedy *et al.*, 2013).

The aim of this study was to evaluate the feasibility of baleen as a novel sample type for assessing patterns in stress-related hormones (cortisol and its metabolites) and reproductive hormones indicative of calving cycles (progesterone and its metabolites). Our specific goals were as follows: (i) to develop methodology for pulverizing baleen and extracting steroids; (ii) to determine whether immunoreactive cortisol and progesterone are detectable in baleen samples; (iii) to validate cortisol and progesterone immunoassays for baleen using standard parallelism and accuracy tests; (iv) to assess patterns of baleen cortisol and progesterone at the base of the baleen plate in relationship to current physiological status (e.g. pregnant vs. non-pregnant females); and (v) to assess hormone variation at different locations along the baleen plate, as a preliminary investigation to determine whether baleen might contain a record of past physiological events.

## Materials and methods

### Baleen samples

**Figure 1:**
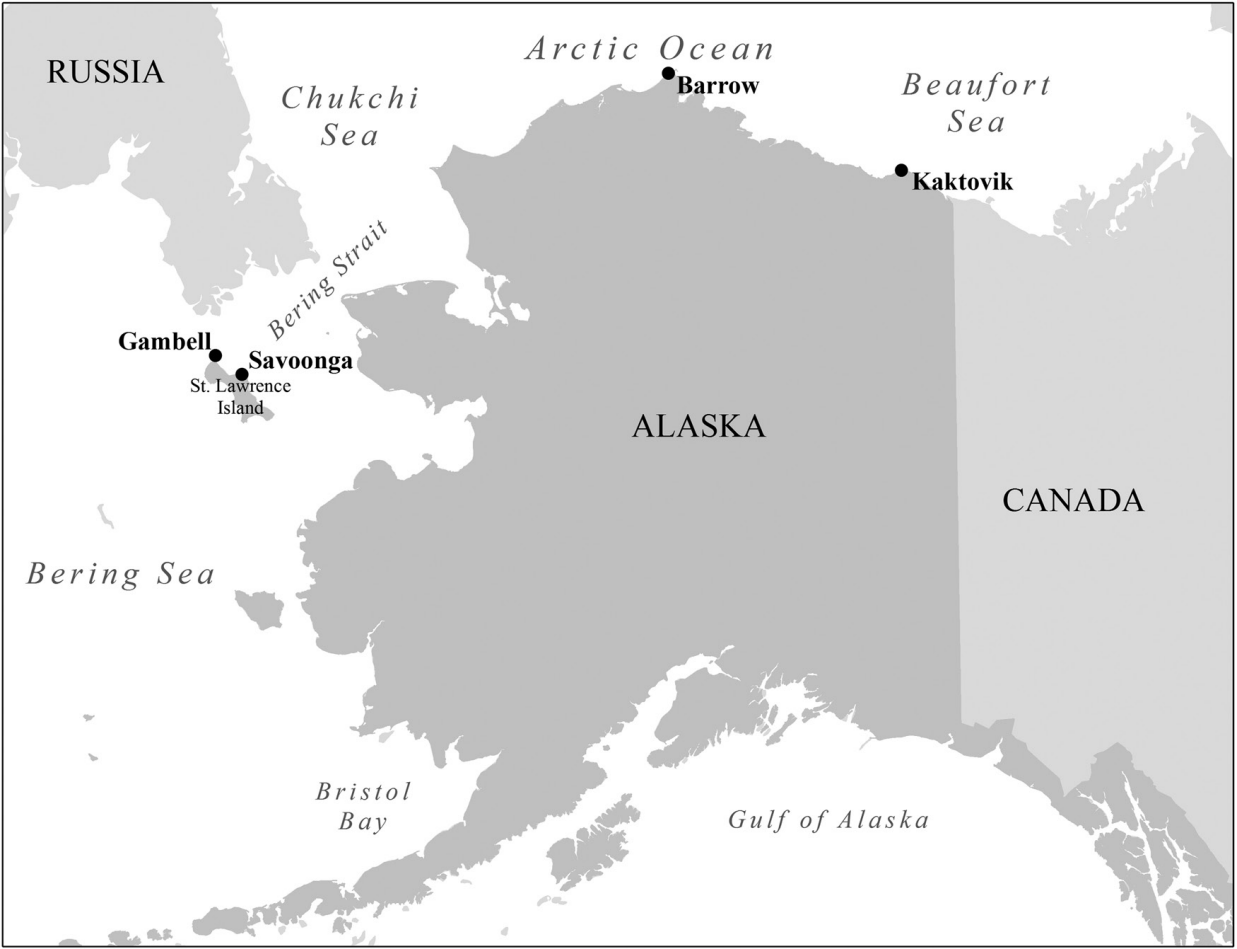
Locations in Alaska at which baleen samples were obtained from landed bowhead whales. Ten whales were sampled at Barrow, three whales at Kaktovik, two at Savoonga and one at Gambell.

Baleen plates were collected between 2003 and 2012 from 16 subsistence-harvested bowhead whales in the towns of Barrow (*n* = 10), Kaktovik (*n* = 3), Savoonga (*n* = 2), and Gambell (*n* = 1), AK, USA (Fig. 1). Sampling was conducted under US National Marine Fisheries Service permit #17350-00. Whales were harvested during spring (*n* = 4) and autumn months (*n* = 12). Baleen plates were stored at ambient temperatures in Barrow (annual temperature range −48 to 26°C, with ∼320 days/year below 0°C) until analysis in 2011. One baleen plate from each whale was sampled, either by cutting a longitudinal strip of ∼40 cm length from the base of the plate (*n* = 3 whales) or by using a DeWalt 18 V cordless power drill equipped with a circular hole saw to excise a disc of baleen ∼2 cm in diameter (*n* = 13 whales). Note that 2 cm represents ∼1.2–1.6 months of baleen growth (Lubetkin *et al.*, 2008). For all whales, baleen was sampled as close to the base of the plate as possible (i.e. most recently grown baleen), but samples could not always be taken precisely from the base of the plate due to fraying of baleen at the base. In some samples, a thin dried layer of fibrous, non-baleen tissue was adhered to the base of the baleen plate; this tissue is lighter in colour than baleen and was discarded whenever noted, but it is possible that some samples contain a small contribution from non-baleen tissue.

**Figure 2:**
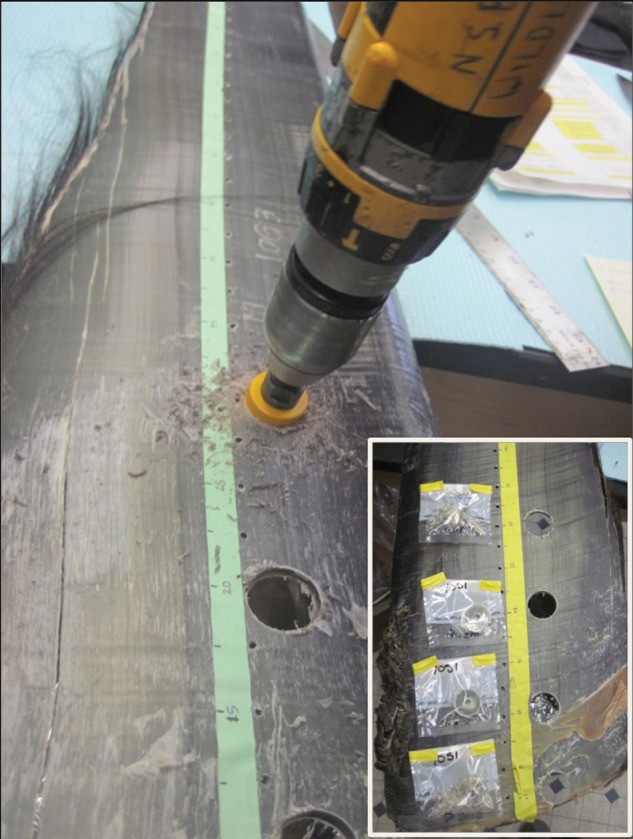
Sampling of a baleen plate from a bowhead whale (*Balaena mysticetus*). A DeWalt 18 V cordless power drill equipped with a circular hole saw was used to excise discs of baleen from four locations: the base of the plate (bottom of photograph; most recently grown baleen) and at 10, 20 and 30 cm distances from the base (i.e. progressively older baleen).

For a subset of whales (*n* = 11), additional discs were excised with the drill centred at 10, 20 and 30 cm distal to the base (Fig. 2). This incremental sampling along the baleen plate was carried out longitudinally (i.e. along the growth axis) as a preliminary investigation into temporal variation in hormone content. These distances (base to 30 cm) represent ∼1.6–2.0 years of baleen growth for most animals in our study (animals >10 m body length; see Lubetkin *et al.*, 2012) and were selected to capture a potential endocrine record of current pregnancies as well as pregnancies of the year before. Given the known variation in baleen growth rate in bowheads, and given the 2 cm diameter of our samples, the four baleen sampling locations are most likely to correlate to time of growth as follows: the ‘base’ sample represents baleen grown between 0.0 and 1.2 months before death; the ‘10 cm’ location represents baleen grown between 6.6 and 7.2 months before death; the ‘20 cm’ location, between 12.6 and 15.2 months before death; and the ‘30 cm’ location, between 18.6 and 23.2 months before death (ranges are based on 2 cm disc diameter and on known baleen growth rate data for bowheads of >10 m body length; Lubetkin *et al.*, 2008; N. Lysiak, personal communication). One animal in our study was <10 m body length, a male of 7.8 m body length; this individual may have had a faster baleen growth rate of up to 25 cm/year (Lubetkin *et al.*, 2008).

The final sample size was 49 subsamples from 16 baleen plates (Table 1). All subsamples (i.e. strips and discs) were shipped to our laboratory at the New England Aquarium (Boston, MA, USA) for analysis.

**Table 1:** Baleen cortisol and progesterone data for 16 bowhead whales

Whale ID	Season of harvest	Length (m)	Sex	Age class	Pregnant?	Cortisol (ng/g)				Progesterone (ng/g)			
						0 cm	10 cm	20 cm	30 cm	0 cm	10 cm	20 cm	30 cm
10G3	Spring	7.8	M	Immature	(male)	0.8				3.9			
06B17	Autumn	10.5	M	Immature	(male)	0.8				4.9			
06B21	Autumn	12.8	M	Immature	(male)	1.4	0.7	0.7	1.1	8.1	3.6	2.6	4.4
06B6	Autumn	13.3	M	Immature	(male)	0.6	0.8	1.1	0.8	4.7	4.5	4.4	4.8
12KK1	Autumn	13.4	M	Immature	(male)	0.9	0.7	0.7	0.6	7.8	2.8	3.0	1.1
06KK3	Autumn	13.7	M	Mature	(male)	1.1	0.7	0.7	0.9	9.0	5.4	5.5	6.8
06B15	Autumn	10.1	F	Immature	No	1.2	0.6	0.7	0.7	12.1	1.6	2.1	1.5
03B15	Autumn	12.5	F	Immature	No	1.8	0.9	0.7	0.9	12.8	3.4	3.3	2.5
05B25	Autumn	13.2	F	Immature	No	1.7				7.6			
08B14	Autumn	13.6	F	Mature	No	1.3	0.7	0.5	0.7	3.3	22.7	3.0	6.5
04B9	Autumn	14.9	F	Mature	No	1.3	0.7	1.0	0.8	9.3	27.7	7.7	246.5
10S3	Autumn	15.5	F	Mature	No	2.4	1.0	1.0	0.6	16.5	188.2	742.0	2143.3
07B12	Spring	14.8	F	Mature	Yes; 31 cm fetus	2.1				23.5			
09KK1	Autumn	15.3	F	Mature	Yes; 163 cm fetus	2.0				444.2			
10S1	Spring	15.0	F	Mature	Yes; 420 cm fetus	2.3	0.6	0.5	0.5	1340.6	759.6	1213.6	2.7
11B3	Spring	17.5	F	Mature	Yes^a^	1.2	0.7	0.7	0.7	481.3	219.2	2.8	4.3

Abbreviations: F, female; M, male. The lengths 0, 10, 20 and 0 cm are distances from the base of the baleen plate; 0 cm is the most recently grown baleen and 30 cm the oldest baleen tested.

^a^11B3 categorized as pregnant based on presence of large corpus luteum and enlarged uterus; however, abdomen was not opened at time of harvest, abdominal organs were frozen the next day, and presence of a fetus could not be confirmed.

### Sex and maturity

Baleen plates were from six males and 10 females (Table 1). Four of the females were pregnant at the time of harvest. Sexual maturity and reproductive state were defined on the basis of body length and relevant anatomical findings at harvest (i.e. testicle size, ovarian morphology), as follows: immature males <13.5 m body length; mature males >13.5 m; immature females <13.5 m; mature females >13.5 m or large ovarian follicles present; and pregnant females were identified by presence of a fetus and/or placenta at harvest, or (if uterus not opened) large corpus luteum and enlarged uterus (Koski *et al.*, 1993; George *et al.*, 1999; O'Hara *et al.*, 2002). Final sample sizes were five immature males (the largest three of which were likely pubertal; O'Hara *et al.*, 2002), one mature male, three immature females, three mature non-pregnant females and four pregnant females (Table 1).

### Baleen sample preparation and steroid extraction

Sample preparation and extraction methods were initially tested on a single baleen plate from a North Atlantic right whale (NARW, *Eubalaena glacialis*). Isopropanol rinses (e.g. Davenport *et al.*, 2006; Ashley *et al.*, 2011; Bechshøft *et al.*, 2011) were not used, because pilot trials showed that such rinses had no effect on apparent cortisol content of NARW baleen (see Supplementary Information).

We tested the following nine different methods of pulverization on NARW baleen: (i) hand-mincing with scissors (following Davenport *et al.*, 2006); (ii) mortar and pestle; (iii) mortar and pestle with liquid nitrogen; (iv) hand-mincing followed by mortar and pestle; (v) commercial blade grinder; (vi) commercial tobacco grinder; (vii) commercial spice grinder; (viii) laboratory tissue homogenizer (MP Biomedicals FastPrep 24); and (ix) rotary electric grinder (Dremel Model 395 Type 5) with a flexible hand-held extension and tungsten-carbide ball tip. The Dremel produced the finest baleen powder with the highest apparent cortisol concentration (see Supplementary Information), indicating superior extraction of hormones. Subsequently, all bowhead whale baleen samples were pulverized with the Dremel grinder, with the resulting baleen powder collected on a weigh paper underneath. For the 2-cm-diameter baleen discs, several areas around the disc were pulverized to obtain a representative sample; for the 40-cm-long strips, a 2-cm-diameter area at the base was pulverized.

Pilot trials on 12 bowhead samples indicated that 50 mg samples of baleen powder sometimes had undetectably low cortisol, whereas a 100 mg sample mass always had detectable cortisol (see Supplementary Information). Therefore, 100 mg of well-mixed baleen powder from each sample was weighed to the nearest 0.0001 g, poured into a 16 mm × 100 mm borosilicate glass tube, capped, and stored at room temperature until extraction within 2 weeks.

### Steroid extraction from baleen

Extraction of steroids from baleen powder followed a consensus protocol derived from the hair and feather glucocorticoid literature (e.g. Davenport *et al.*, 2006; Bortolotti *et al.*, 2008). Briefly, 4.0 ml of 100% methanol was added to 100 mg of baleen powder, vortexed for 20 h at room temperature and centrifuged for 15 min at 4000*g*. The supernatant was pipetted into a separate borosilicate glass tube for drying, and the pellet was rinsed twice more to recover additional hormone. For each rinse, 1.0 ml of 100% methanol was added to the pellet, vortexed for 30 s, centrifuged for 15 min at 4000*g*, and the supernatant was transferred to the drying tube. The combined supernatant was dried under air blow in a ThermoScientific Reacti-Therm III set at 45°C. When the volume reached <0.5 ml, another 1.0 ml methanol was added to rinse down the walls of the tube, and the sample was again dried. Once fully dry, samples were reconstituted in 0.5 ml of assay buffer from the progesterone assay kit, transferred to a cryovial and stored at −20°C until assay within 1 week of extraction.

### Assay validations

We tested parallelism and accuracy of bowhead whale baleen extracts using a cortisol enzyme immunoassay (EIA; catalogue #K003-H1) and a progesterone EIA (catalogue #K025-H1), both from Arbor Assays (Ann Arbor, MI, USA). These two assays were selected based on previous successful use with faeces and respiratory vapour from other baleen whales. Cortisol rather than corticosterone was tested based on evidence that cortisol is probably the major circulating glucocorticoid of baleen whales (R. Rolland, unpublished data; Birukawa *et al.*, 2005). The manufacturer's reported assay specifications are as follows: for the cortisol assay, sensitivity = 17.3 pg/ml, limit of detection = 45.4 pg/ml, intra-assay precision = 8.8%, inter-assay precision = 8.1%; cross-reactivities, dexamethasone = 18.8%, prednisolone = 7.8%, corticosterone = 1.2%, cortisone = 1.2% and all other tested steroids <0.1%; and for the progesterone assay, sensitivity = 47.9 pg/ml, limit of detection = 52.9 pg/ml, intra-assay precision = 3.2%, intra-assay precision = 5.7%; cross-reactivities, 3β-OH-progesterone = 172%, 3α-OH-progesterone = 188%, 11β-OH-progesterone = 2.7%, 11α-OH-progesterone = 147%, 5α-dihydroprogesterone = 7.0%, pregnenolone = 5.9% and all other tested steroids <0.1%. This progesterone assay uses the antibody previously reported in faecal hormone literature as ‘CL#425’ (e.g. Rolland *et al.*, 2005). For both assays, the manufacturer's protocol was used except that standards for the cortisol assay were made with the progesterone assay buffer, based on technical advice from the manufacturer.

Progesterone assay parallelism was tested using three separate baleen extract pools from pregnant females, non-pregnant females and males. These three pools were serially diluted in assay buffer and assayed alongside known-dose progesterone standards. Due to low cortisol content in most baleen samples, cortisol parallelism was tested using a single pool from samples containing the highest cortisol content in a pilot assay. This ‘high-cortisol’ pool was serially diluted in buffer and assayed alongside the cortisol standard curve. Results were plotted as the percentage bound vs. the logarithm of relative dose, and the slopes were compared for parallelism of the linear portion of the curve. Based on parallelism results, all subsequent samples were assayed at 1:4 for progesterone and at 1:1 (full-strength extract) for cortisol; these dilutions were chosen to fall near the 50% bound on the standard curve, the area of greatest assay precision.

Assay accuracy was assessed by spiking standard curves with an equal volume of a ‘low-progesterone’ pool (samples that had low progesterone in a pilot assay) diluted to 1:4, or a ‘low-cortisol’ pool (samples that had low cortisol in a pilot assay) at 1:1, and assaying alongside standards spiked only with assay buffer. Results were plotted as the observed dose vs. known standard dose and assessed for linearity, slope and *y*-intercept.

All assays were performed in duplicate, with a full standard curve in each assay, non-specific binding wells in quadruplicate and ‘zero’ (blank) wells in quadruplicate. Any samples with >10% coefficient of variation between duplicates, or with the percentage bound <10 or <90%, were rediluted accordingly and re-assayed. Multiple samples from the same whale (e.g. base, 10, 20 and 30 cm samples) were assayed simultaneously in the same EIA plate, except for an initial set of 12 base samples that were assayed on a separate EIA plate.

### Statistical analysis

Data were analysed using InStat 3.0b for Macintosh OSX (GraphPad Software Inc., San Diego, CA, USA). Assay parallelism was assessed with *F*-tests on data for the linear portion of the curves. Assay accuracy was assessed as follows: (i) goodness-of-fit of linear regression line on expected vs. observed dose (e.g. *r*^2^ should ideally be >0.90); (ii) slope within 0.7–1.3; and (iii) good match of *y*-intercept to the apparent dose when the pool was assayed alone. Cortisol data were normally distributed and therefore analysed with parametric tests (Zar, 2009). Progesterone data were highly skewed and could not be normalized with common transformations, and were analysed with non-parametric tests (Conover, 1999). Differences between males vs. females and non-pregnant vs. pregnant females were analysed with Student's *t*-tests or Mann–Whitney *U*-tests. Patterns in hormones at different locations along a baleen plate (base, 0, 20 and 30 cm) were analysed with repeated-measures ANOVA (with Tukey–Kramer *post hoc* tests) or Kruskal–Wallis tests. Data are presented as means ± SEM for normally distributed data or medians for non-normal data. The significance level was set at α = 0.05.

## Results

### Assay validations

**Figure 3:**
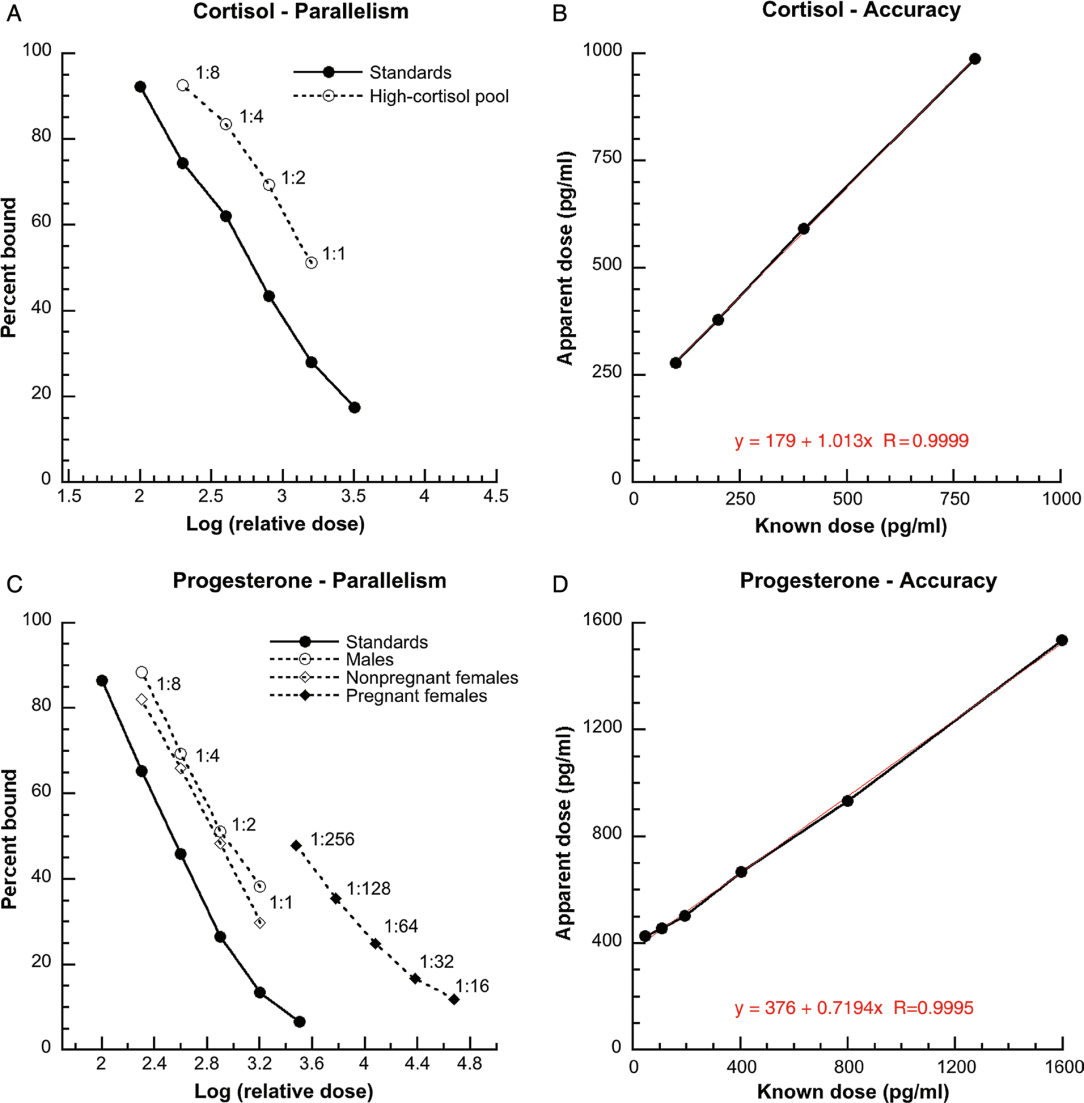
Validation results for pooled baleen extracts from bowhead whales for cortisol (top) and progesterone (bottom). Note close parallelism of the serially diluted samples to the standard curves (**A** and **C**; linear portions of curves shown), and accuracy graphs that are relatively straight, with slopes close to 1.0 (**B** and **D**; equation of best-fit linear regression line shown).

Both assays demonstrated good parallelism and accuracy for bowhead baleen extracts. In the parallelism tests, the slope of the serially diluted baleen pool(s) was not significantly different from the slope of the standard curve (for progesterone, male pool, *F*_1,6_ = 0.0359, *P* = 0.856; non-pregnant female pool, *F*_1,6_ = 0.2030, *P* = 0.6681; and pregnant female pool, *F*_1,5_ = 1.756, *P* = 0.2424; and for cortisol, *F*_1,6_ = 1.0022, *P* = 0.3554; Fig. 3). In the accuracy tests, the slope of observed vs. expected concentration was straight and was within 0.7–1.3 for both assays, and there was a close fit between the *y*-intercept and the observed dose of the pool alone (progesterone, *r*^2^ = 0.9989, slope = 0.7194, *y*-intercept = 376 pg/ml and pool = 424 pg/ml; and cortisol, *r*^2^ = 0.9998, slope = 1.013, *y*-intercept = 179 pg/ml and pool = 205 pg/ml; Fig. 3).

### Baleen cortisol

**Figure 4:**
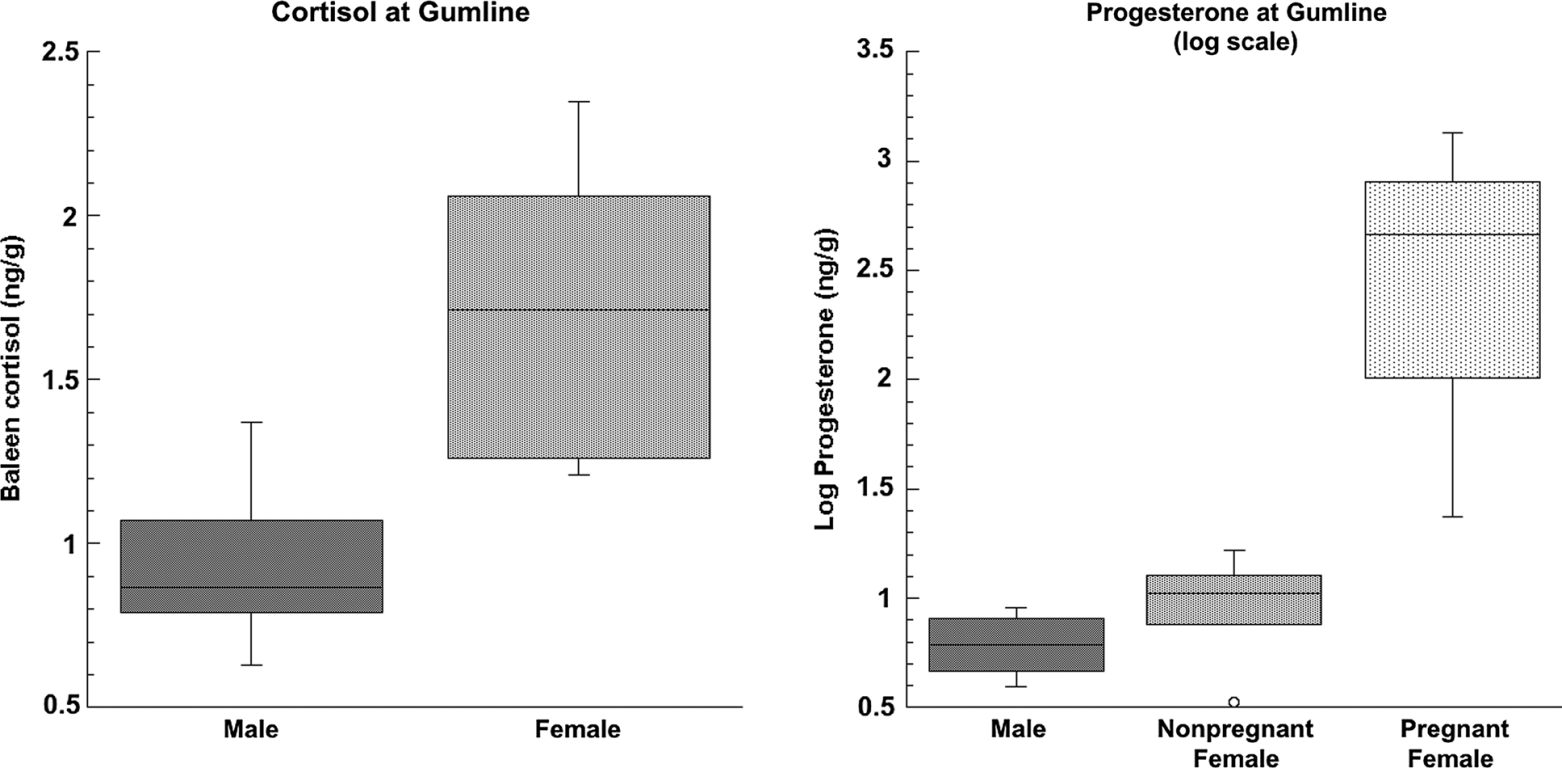
Boxplots of baleen cortisol and baleen progesterone at the base of the plate (recently grown baleen) in male (*n* = 6) and female bowhead whales (*n* = 6 nonpregnant and *n* = 4 pregnant, total 10). Note that progesterone is shown on a logarithmic scale.

Cortisol (or immunoreactive cortisol metabolites) was detectable in all baleen samples (Table 1). In base samples, females had significantly higher cortisol than males (females, mean = 1.72 ± 0.14 ng/g; males, mean = 0.93 ± 0.11 ng/g; *t*_14_ = 3.819, *P* = 0.0019; Fig. 4). This difference disappeared if pregnant females were excluded (mature males vs. mature nonpregnant females, *t*_5_ = 1.777, *P* = 0.1358). Pregnant females tended to have higher cortisol than non-pregnant females (Table 1), although this difference was not significant (*t*_8_ = 1.1263, *P* = 0.2927), but note that statistical power for this test was low.

Cortisol content was significantly higher in the base samples than in three distal samples (older baleen at 10, 20 and 30 cm), but the distal samples from the same animals were not significantly different from each other (within-individual; *F*_3,10_ = 12.166, *P* < 0.0001; *P* < 0.05 for *post hoc* comparisons of base vs. any other location; Table 1). Furthermore, in contrast to the sex difference seen in the base samples, males and females did not exhibit significant differences in cortisol at the 10, 20 or 30 cm locations.

### Baleen progesterone

**Figure 5:**
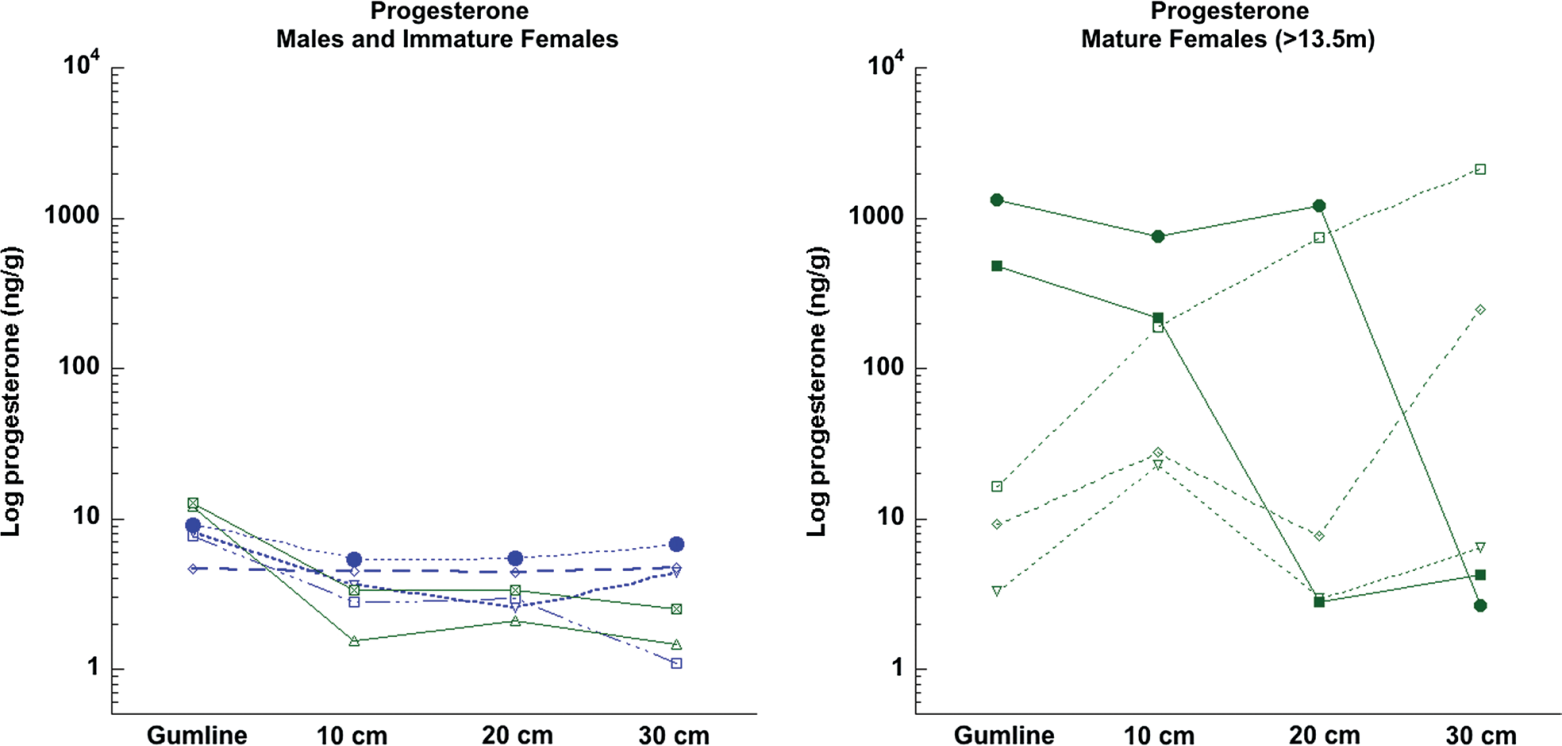
Baleen progesterone at four different locations along baleen plates from bowhead whales (*n* = 11). Left panel, males (blue) and immature females (green); the single mature male is indicated with filled blue circles. Right panel, mature females; continuous lines and filled symbols indicate two females that were pregnant at the time of harvest; dotted lines and open symbols indicate females that were not pregnant at harvest. Note that two of the nonpregnant females have elevated progesterone concentrations in older baleen, possibly indicating prior pregnancy. The base location is the most recently grown baleen; the 10, 20 and 30 cm locations (i.e. centimetres from the base) represent progressively older baleen. Note the logarithmic scale on the *y*-axis.

Progesterone (or immunoreactive progesterone metabolites) was detectable in all baleen samples (Table 1). Similar to cortisol, females had significantly higher base progesterone than males, and this difference disappeared if pregnant females were excluded (females, median = 14.65 ng/g; males, median = 6.33 ng/g; *U* = 9.000, *P* = 0.023; pregnant females excluded and mature animals only, *t*_5_ = 0.6908, *P* = 0.5204; Fig. 5). All pregnant females had higher progesterone in the base sample than all non-pregnant females, and this difference was significant (*U* = 24.000, *P* = 0.0095).

In the baleen plates that were subsampled at multiple locations, all four males and both immature females exhibited consistently low progesterone (<20 ng/g) at all locations (Table 1 and Fig. 5). These six animals had slightly, but significantly, higher progesterone at the base location compared with the other three locations (*F*_3,5_ = 11.007, *P* = 0.0004; Fig. 5). In contrast, four of five mature females showed extreme variation in progesterone, spanning one to three orders of magnitude within a single individual, at different locations along the baleen plate (Table 1 and Fig. 5). Mature females exhibited no relationship of progesterone with baleen location (*Fr*_5,4_ = 2.040, *P* = 0.6522), and progesterone was occasionally very high even in the oldest baleen tested (Table 1). In the two pregnant females that were tested at multiple baleen locations (whales 10S1 and 11B3), both had extremely high progesterone at the base and 10 cm locations, and the smaller whale (10S^1^) also had very high progesterone at the 20 cm location.

## Discussion

Our results indicate that immunoreactive cortisol and progesterone are measurable in bowhead whale baleen using commercially available immunoassay kits. Both hormones appear to reflect physiological parameters of interest, such as sex and reproductive state, with baleen progesterone exhibiting variation consistent with occurrence of past pregnancies. To our knowledge, this is the first demonstration of successful measurement of steroid hormones in baleen from any species of whale.

### Baleen cortisol

The higher baleen cortisol levels found in females compared with males, at the base of the plate, is reminiscent of sex differences in circulating glucocorticoids seen in many mammals (Tilbrook *et al.*, 2000; Panagiotakopoulos and Neigh, 2014). Sex differences in cortisol have been documented in other cornified tissues as well (e.g. polar bear hair, Bechshøft *et al.*, 2011; Macbeth *et al.*, 2012; human hair, Dettenborn *et al.*, 2012). In the present study, the highest baleen cortisol levels detected were in pregnant females. Female mammals commonly have elevated glucocorticoids during pregnancy (Foley *et al.*, 2001; D'Anna-Hernandez *et al.*, 2011), and a similar pattern has been documented in faecal glucocorticoids of North Atlantic right whales (Hunt *et al.*, 2006). Although preliminary, our results indicate that baleen, at least at the base, may preserve a hormonal signature of adrenal activity in bowhead whales.

Cortisol concentrations were markedly lower in samples taken from older baleen (10, 20 and 30 cm) compared with base samples. This pattern was also noted in progesterone data, but to a lesser degree. Cortisol is one of the most polar steroids, whereas progesterone is relatively non-polar; it may be that cortisol is more likely to leach from baleen into seawater. Human hair loses some cortisol upon immersion in hot water (Li, 2012), and it is unknown whether a similar process might occur in baleen immersed in cold seawater over months. However, any such leaching does not seem to continue beyond 10 cm, because the 10, 20 and 30 cm samples had similar cortisol concentrations. If loss of hormone to seawater does occur, baleen from the core of the plate may be protected from any such loss. Our samples consisted of a mix of surface and interior baleen; future research could compare surface vs. core baleen separately. Additionally, assay of other adrenal steroids that are less polar than cortisol, such as corticosterone and aldosterone, may prove fruitful for distinguishing periods of higher adrenal activity in older baleen.

### Baleen progesterone

The dramatic variation in baleen progesterone profiles of the mature females suggests that baleen may contain an endocrine record of pregnancy. The progesterone profiles seen in individual females are consistent with estimates of bowhead gestation length and baleen growth rate (Koski *et al.*, 1993; Lubetkin *et al.*, 2008). Baleen growth rate in bowheads slows with age; for the animals sampled in this study, baleen growth rate was estimated at ∼19–20 cm/year for the smaller, younger females and ∼17–18 cm/year for the largest and oldest female (Lubetkin *et al.*, 2008, 2012; C. George, personal observation; N. Lysiak, personal communication). For example, in the smaller pregnant female, 10S1, the 20 cm sample was probably grown ∼12–13 months prior to death, while in the larger pregnant female, 11B3, the 20 cm sample was probably grown ∼13.5–15 months prior to death. Given an estimated gestation length of 13–14 months (Koski *et al.*, 1993), it therefore makes sense that the smaller female had high progesterone that spanned three locations (base, 10 and 20 cm), i.e. her 20 cm sample was probably grown during pregnancy. In contrast, the larger female had high progesterone that spanned only two locations (base and 10 cm, but not 20 cm); thus, her 20 cm sample may represent pre-pregnancy. Although these patterns are preliminary, they suggest that baleen may retain an endocrine signature of prior pregnancies and that baleen should be investigated further as a potential retrospective record of past reproductive cycles.

We note, however, that there was one pregnant female (07B12; Table 1) that had much lower base progesterone than the other pregnant females (though still higher than all non-pregnant females; Table 1). This female was in early pregnancy, as determined by small fetal size (C. George, personal observation). Lower progesterone levels might be characteristic of early pregnancy. Alternatively, it is possible that the base sample of this female may not have been taken precisely from the growth zone, due to fraying at the base of the plate (see Materials and methods) and may represent pre-pregnancy.

Overall, these data indicate that baleen progesterone should be investigated further as a retrospective record of prior reproductive cycles in bowhead whales. Baleen hormone data could be coupled with ovarian analysis (e.g. corpora-albicans counts; George *et al.*, 2011) and may provide an alternative, and much-needed, method of estimating inter-calving intervals and reproductive cycles in baleen whales. Sampling at more locations along the baleen plate, ideally coupled with stable isotope analysis to discern annual cycles (Lubetkin *et al.*, 2008), could help match adrenal and reproductive hormone profiles to season and year.

### Future methodological research

The processing and extraction methods presented here provide an introduction to the technique, but should not be regarded as a final protocol. Future experiments should include tests of other extraction methods (e.g. methanol vs. ethanol, vortex duration, extraction temperature), with the goal of maximizing extraction of steroids from baleen powder. High-performance liquid chromatography or related techniques could be used to confirm the chemical identity of steroids present in baleen, as it is possible that immunoreactive steroids detected by EIA antibodies are not necessarily pure parent hormones; unusual tissue types sometimes contain uncommon steroid metabolites and/or conjugated steroids (Wasser *et al.*, 1988). Furthermore, additional antibodies and other quantification methods should be tested. Other steroids could be tested as well, particularly testosterone, estradiol, aldosterone and corticosterone.

Variation in hormone content in different sections of the baleen plate should also be investigated, e.g. lingual vs. labial edge, dorsal surface vs. ventral surface and surface vs. core of plate. Potential contributions from any non-baleen dried tissue that adheres to the sample should be evaluated, as should variation between plates from different locations in the mouth (left vs. right side, anterior vs. posterior plates).

### Conclusions

In conclusion, baleen steroid analysis represents a new tool that shows great potential for retrospective assessment of patterns of physiological stress and reproductive cycles of baleen whales. Of particular interest is the availability of historical baleen samples in museum archives (samples collected during the era of commercial whaling) that could be used for comparisons with present-day population data. Additionally, if this method proves reliable, continued collection of baleen from modern populations may allow population changes to be tracked through time (e.g. potential shifts in average inter-calving intervals), as well as assessment of potential impacts of ongoing climate change and increasing human activity in the Arctic. In sum, hormone analysis of baleen could provide an innovative means to evaluate long-term trends of stress and reproduction in whale populations exposed to a changing marine environment.

## Supplementary material

Supplementary material is available at *Conservation Physiology* online.

## Funding

This work was supported by the NSB [contract #2012-165 to the New England Aquarium] with funding from the NSB/Shell Baseline Studies Program.

## Supplementary Material

Supplementary Data
