# Supplementary material for: Baleen hormones: a novel tool for retrospective assessment of stress and reproduction in bowhead whales (Balaena mysticetus)
Source: Conserv Physiol. 2014 Aug 12;2(1):cou030. doi: 10.1093/conphys/cou030 (PMC4806734; doi:10.1093/conphys/cou030)
Supplement: Supplementary Data [file supp_2_1_cou030__index.html]

Supplementary Data 

# Baleen hormones: a novel tool for retrospective assessment of stress and reproduction in bowhead whales (*Balaena mysticetus*)

## Supplementary Data

Supplementary Data

**Files in this Data Supplement:**

- Supplementary Data - Docx file
- Supplementary Figure 1 - jpg file
