# Supplementary material for: Baleen hormones: a novel tool for retrospective assessment of stress and reproduction in bowhead whales (Balaena mysticetus)
Source: Conserv Physiol. 2014 Aug 12;2(1):cou030. doi: 10.1093/conphys/cou030 (PMC4806734; doi:10.1093/conphys/cou030)
Supplement: Supplementary Data [file supp_cou030_cou030supp.docx]

Baleen hormones: A novel tool for retrospective assessment of stress and reproduction in bowhead whales (*Balaena mysticetus*)

SUPPLEMENTARY INFORMATION

*Pulverization Methods*

Most of the published studies measuring glucocorticoids in hair samples utilize a scissor-mincing method to process the samples for extraction, following the work of Davenport et al. (2006). In contrast, stable-isotope testing on baleen often uses electric drills, mills or grinders to pulverize baleen to a powder (e.g. Lubetkin 2008, Ryan et al., 2013), but it is unknown whether the heat produced by these methods might degrade immunoreactive hormones. We tested scissor mincing, electric grinders and a variety of other pulverization methods using a single baleen plate from a North Atlantic right whale (NARW, *Eubalaena glacialis)*. Nine methods were initially compared for their ability to pulverize NARW baleen to small particle size:

(1) "Scissors": hand-mincing with stainless-steel surgical scissors

(2) #1 followed by commercial stainless-steel blade grinder (coffee grinder)

(3) #1 followed by commercial stainless-steel tobacco grinder

(4) #1 followed by commercial stainless-steel peppercorn grinder

(5) hand-grinding with mortar-and-pestle alone

(6) #1 followed by mortar-and-pestle

(7) "LN2": #1 followed by mortar-and-pestle containing liquid nitrogen

(8) #1 followed by laboratory tissue homogenizer (MP Biomedicals FastPrep 24), tested with both ceramic balls and garnet chips

(9) "Dremel": Rotary electric grinder (Dremel Model 395 Type 5) fitted with a tungsten-carbide cylindrical tip

Only three methods were able to reduce baleen particle size at all: scissors (method #1), reduced baleen to ~1-3mm particles, LN2 (method #7) further reduced the scissor-minced particles to ~1mm particles, and method #9 (Dremel) produced a consistent fine powder with particle size estimated (via microscope measurements) at ~0.1-0.01mm diameter. Particle size of scissors method (#1) and Dremel method (#9) are compared in Supplementary Figure 1.

No other methods appeared to affect baleen particle size at all. Generally, baleen pieces are both strong and flexible, and were resistant to methods commonly used to pulverize other biological tissues. For example, method #8 (laboratory tissue homogenizer with ceramic beads and garnet chips) not only did not affect baleen particle size at all, but rather the homogenizer's pulverization materials were themselves pulverized. That is, instead of the garnet chips pulverizing the baleen, the baleen reduced the garnet chips to a fine garnet dust.

We compared the three best methods - Dremel, scissor-mincing, and LN2 - for apparent cortisol content using several baleen subsamples taken from within 1cm of gumline of a baleen plate from a pregnant North Atlantic right whale (NARW). For this pilot trial, 50mg of each pulverized subsample (n=4 Dremel samples, n=1 LN2 sample, n=3 scissor-minced samples) was extracted with 4.0mL 100% methanol, vortexed for 20 hours, centrifuged at 3000rpm for 15min, and the methanol pipetted to a dry-down tube. Of the four Dremel samples, two were from an area of baleen that had been rinsed with isopropanol, and two were not (see below); these four subsamples are combined in this analysis. Only 1 LN2 sample was tested due to limited sample available from the gumline. Each sample was extracted as described in the Methods, reconstituted in Arbor Assays cortisol buffer and assayed with cortisol EIA #K003-H1 (Arbor Assays, Ann Arbor, MI). The four Dremel samples had apparent cortisol of 217, 275, 311, and 400 pg/ml (mean± SEM = 301 ± 38 pg/ml); the three scissor-minced samples had mean cortisol of 72, 83, and 92 pg/ml (mean ± SEM = 82 ± 6 pg/ml); and the single LN2 sample had cortisol of 89 pg/ml. Overall, the Dremel samples had, on average, 3.38x higher cortisol than the LN2 sample, and 3.66x higher cortisol than scissor-minced samples from the same area of the same baleen plate. We attribute this difference to superior hormone extraction from Dremel samples, due to their finer particle size. Therefore, we used the Dremel pulverization method for all subsequent samples.

**Rinse testing with NARW baleen**

A literature search on published methods for extracting glucocorticoids from keratinized substrates (hair, feathers) revealed that hair samples are typically rinsed or washed in an alcohol bath (typically isopropanol) before extraction, in order to remove possible surface contamination, e.g. from blood (Davenport et al. 2006, Ashley et al. 2011, Bechshøft et al. 2011). In contrast, feather samples typically are not rinsed (Bortolotti et al. 2008, Lattin *et al.* 2011). However, the costs and benefits of rinsing have not been adequately investigated for either type of sample, and there remain questions about whether such rinsing might strip native hormone from the sample. As a preliminary investigation into this issue for baleen, we compared cortisol content of two rinsed vs. two un-rinsed samples from the gumline of the same baleen plate from a pregnant female NARW. All samples were pulverized with Dremel. Two subsamples were taken from the gumline before rinsing the baleen plate. We then performed two brief rinses with 100% isopropanol, dripping ~5mL briefly (~15 sec) over a small 5cm-wide section of the gumline, letting the isopropanol drip to the sink, repeating the process (second rinse), patting the plate dry with a Kimwipe and then letting it air-dry. Two more gumline samples were then taken from the rinsed area, and extracted and assayed as described above. The two unrinsed samples had apparent cortisol of 311 and 217 pg/ml; the two rinsed samples had cortisol of 275 and 400 pg/ml. Based on this very small pilot test, rinsing with isopropanol appeared to have no detectable effect at reducing hormone level due to any surface contamination; or at least, any such effect is within the range of normal variation. We emphasize that this was a very small pilot trial only and this question should be tested further, and we further point out that variation in hormone content at different regions on a baleen plate must be investigated as well (see Discussion).

**SUPPLEMENTARY FIGURE LEGEND**

**Supplementary Figure 1.** Examples of baleen particle size viewed under light microscope after (left) scissor mincing (left) and (right) Dremel pulverization. Note the much smaller particle size produced by the Dremel. Scale bar = 1mm, magnification = 43.5X.
