# Supplementary figures and images for: Baleen hormones: a novel tool for retrospective assessment of stress and reproduction in bowhead whales (Balaena mysticetus)
Source: Conserv Physiol. 2014 Aug 12;2(1):cou030. doi: 10.1093/conphys/cou030 (PMC4806734; doi:10.1093/conphys/cou030)

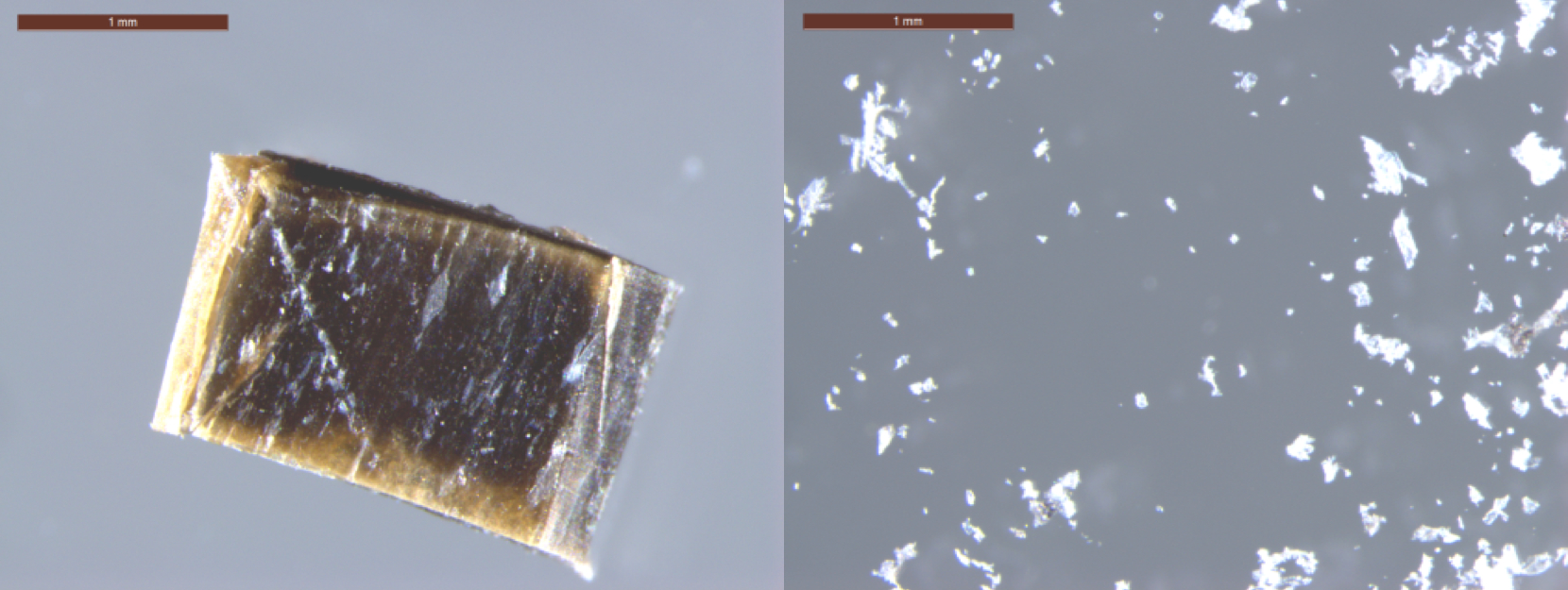

Supplement: Supplementary Data [file supp_cou030_cou030supp_fig1.jpg]
